# P-1583. Can Electronic Clinical Decision Support Systems Decrease Unnecessary Antibiotic use for Asymptomatic Bacteriuria?

**DOI:** 10.1093/ofid/ofae631.1750

**Published:** 2025-01-29

**Authors:** Aoi Yogo, Elie Saade, Brigid Wilson, Timothy C Jenkins, Eric Ransom, Abhishek Deshpande, Curtis Donskey, Zainab Albar, Lauren H Epstein, Leila S Hojat

**Affiliations:** Case Western Reserve University, Cleveland, Ohio; Case Western Reserve University, Cleveland, Ohio; VA Northeast Ohio Healthcare System, Cleveland, Ohio; Denver Health, Denver, Colorado; Case Western Reserve University, Cleveland, Ohio; Cleveland Clinic, Cleveland, Ohio; Cleveland VA Hospital, Cleveland, Ohio; Case Western Reserve University, Cleveland, Ohio; Emory University School of Medicine, Atlanta, GA; Case Western Reserve University/ University Hospitals Cleveland Medical Center, Cleveland, OH

## Abstract

**Background:**

Asymptomatic bacteriuria is often treated unnecessarily with antibiotics. Restricting cultures to patients who meet specific urinalysis (UA) parameters has been shown to decrease urine culture rate; however, the impact of these interventions on antibiotic use is not well established. We describe the impact of UA with reflex to culture implementation on urine culture rate and antibiotic use in a large healthcare system.

Figure 1

**Figure:**
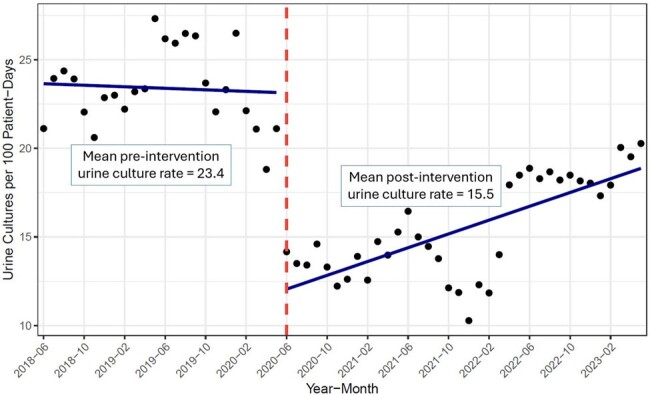

Mean urine culture rate across system pre- and post-intervention. Each point represents the monthly average number of urine cultures per 100 patient-days across the healthcare system.

**Methods:**

A retrospective cohort study was conducted in an integrated healthcare system in Northeast Ohio from June 2018 to May 2023. We initiated an intervention in June 2020 in which urine cultures were reflexively performed only for UA with ≥10 white blood cells, positive leukocyte esterase, and/or positive nitrate (UA with reflex to culture), excluding special populations. We calculated monthly rates of inpatient or emergency department urine cultures and compared average rates pre- and post-intervention. Additionally, we calculated pre- and post-intervention antibiotic use data for adults hospitalized during the study period for whom urine culture was attempted (urine culture or UA with reflex to culture ordered) to compare days of therapy (DOT) per days hospitalized and days of antibiotic spectrum coverage score (DASC) per DOT within 7 days of urine test order.

Table 1

**Figure:**
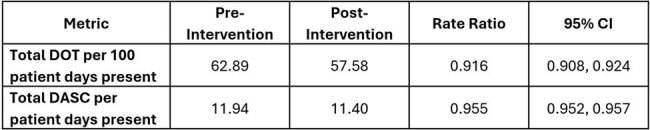

Summary of comparison of antibiotic days of therapy and days of antibiotic spectrum coverage metrics pre- and post-intervention within 7 days of urine test. Abbreviations: DOT, days of therapy; DASC, days of antibiotic spectrum coverage; CI, confidence interval.

**Results:**

A total of 101,337 urine cultures among 8 hospitals met eligibility criteria during the study period. The mean monthly urine culture rate was 23.4 cultures per 100 patient-days pre-intervention and 15.5 post-intervention (mean difference 7.9, 95% confidence interval [CI] 6.6-9.2), with a large initial decrease followed by increasing rates in 2022 (**Figure 1**). Among adults with urine cultures, the post-intervention group had 7.5% lower DOT (rate ratio [RR] 0.916; CI 0.908, 0.924) and 5.1% lower DASC (RR 0.955; CI 0.952, 0.957) within 7 days of culture relative to the pre-intervention group (**Table 1**).

**Conclusion:**

After implementing urine culture CDS in a large health system, hospitalized adult patients had a lower urine culture rate, and DOT and DASC were lower among those for whom urine culture testing was attempted. Further investigation is needed to determine the sustainability of the effects of CDS alone versus a multifaceted intervention and its impact of CDS on clinically relevant outcomes.

**Disclosures:**

**Elie Saade, MD, MPH, FIDSA**, Janssen Global Services: Advisor/Consultant|Janssen Global Services: Advisor/Consultant|Janssen Research and Development: Advisor/Consultant|Janssen Research and Development: Advisor/Consultant **Abhishek Deshpande, MD, PhD**, AHRQ: Grant/Research Support|Clorox Healthcare: Grant/Research Support **Curtis Donskey, MD**, Clorox: Grant/Research Support|Pfizer: Grant/Research Support

